# Concomitant acute right ventricular infarction and ischemic cerebrovascular stroke; possible explanations

**DOI:** 10.1186/1755-7682-3-25

**Published:** 2010-10-26

**Authors:** Hesham R Omar, Ahmed Fathy, Rania Rashad, Engy Helal

**Affiliations:** 1 Department of Cardiovascular Medicine, Cairo University Hospital, Cairo, Egypt; 2 Department of Cardiovascular Medicine, National Heart Institute, Cairo, Egypt; 3 Department of Critical Care, Cairo University Hospital, Cairo, Egypt; 4 Emergency Department, Elagouza Police Hospital. Cairo, Egypt

## Abstract

Concomitant acute myocardial infarction and ischemic cerebrovascular accidents has been rarely reported in the literature. In this report, we are describing a 48 year old male patient who presented with acute infero-posterior and right ventricular transmural myocardial infarction followed within one hour with massive cerebral infarction and deep coma. The patient succumbed to cardiogenic shock and fatal ventricular arrhythmias resistant to aggressive resuscitative efforts. This association can best be described as "cardio-cerebral infarction". The authors suggest that there exist a possible relationship between both pathologies rather than being just a mere coincidence. Explanations for this association are thoroughly explored and discussed. Early recognition of such cases is important and determines the patient's further management and prognosis. This report aims to sensitize readers to this rare and critical scenario and highlights the necessity of further research for the ideal management of this situation.

## Introduction

Myocardial infarction is believed to increase the risk of ischemic cerebrovascular strokes. Most studies had determined an increased incidence of ischemic strokes after anterior myocardial infarction on both short and long term bases. This was attributed in the vast majority of cases to embolization due to left ventricular mural thrombi or atrial fibrillation. However, the relation between ischemic cerebrovascular strokes and acute right ventricular infarction has been rarely discussed. In this report, we are describing a rare case in which right ventricular infarction was followed in no time by cerebrovascular stroke and discussing the possible mechanisms explaining this association.

## Case presentation

A 48 year old male patient presented to the emergency department with a one hour history of epigastric pain, vomiting and profuse sweating. The patient's medical history was significant for long standing hypertension and chronic heavy cigarette smoking as well as recurrent attacks of chest pains related mainly to exertion and relieved by rest.

Clinical examination revealed an obese restless patient with a blood pressure of 90/60 bilaterally and a regular pulse at 110 beats per minute. The jugular veins were congested with a venous pressure of 15 mmHg above the sternal angle and positive Kussmaul's sign. Cardiac examination revealed normal first and second heart sounds and an S3 gallop but no murmurs or pericardial rub. Chest examination revealed bilaterally equal air entry with normal vesicular breathing and no rhonchi or crepitations.

**Figure 1:**
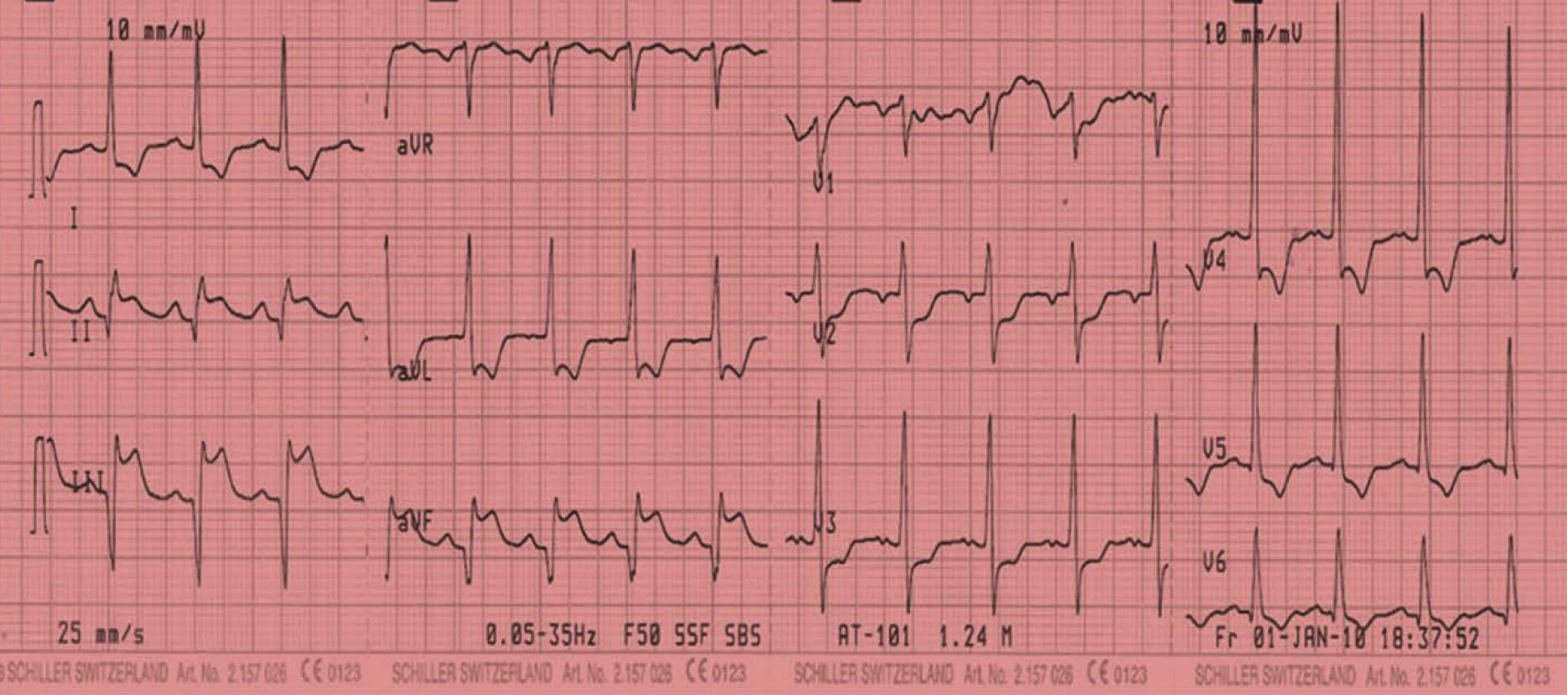
**Revealing pathological Q waves and ST segment elevation in leads II, III, aVF with reciprocal ST segment depression in the lateral leads**.

**Figure 2:**
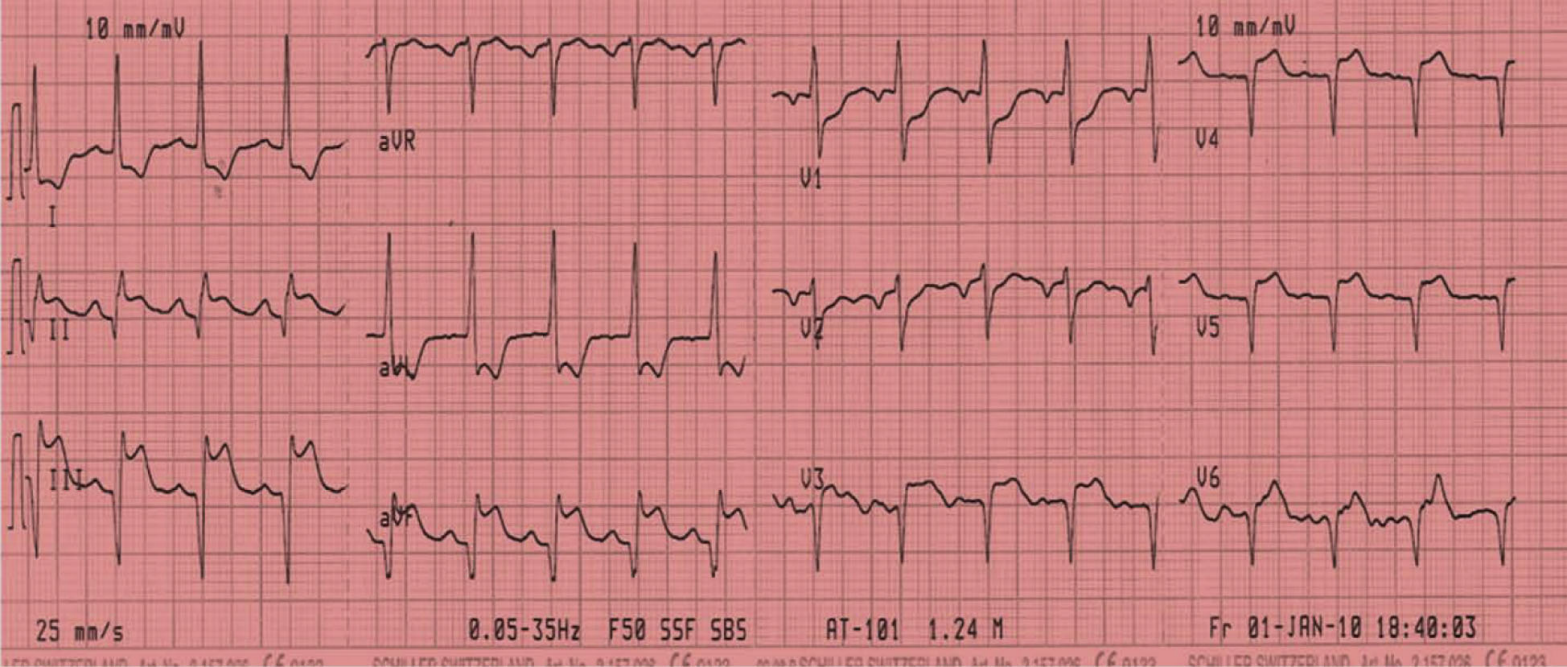
**Right precordial leads revealing ST segment elevation in V3R and V4R denoting right ventricular infarction**.

Electrocardiogram was performed in the emergency room and revealed Q waves and ST segment elevation in the inferior leads as well as tall R wave and ST segment depression in lead V2 as shown in figure 1. Right chest leads showed ST segment elevation in lead V3R and V4R as shown in figure 2. The clinical picture together with electrocardiographic finding suggested a diagnosis of acute transmural infero-posterior associated with right ventricular myocardial infarction.

Immediately after admission to the emergency room, the patient's conscious level ran a downhill course and he went into deep coma with a GCS of 3/15 and pinpoint pupils. A computed tomography of the brain was performed and revealed no abnormalities. This deterioration was presumed to be due to acute ischemic stroke not yet evident in the initial CT scan. Taking into consideration this sudden worsening of his conscious level and a calculated NIHSS score (National Institute of Health Stroke Scale) of >25 indicating a massive cerebral infarction, it was decided not to administer thrombolytic therapy. We also decided not to undergo primary percutaneous transluminal coronary angioplasty because of the bad prognosis of the patient. The patient was treated conservatively with antiplatelets, anticoagulant therapy and inotropic support for cardiogenic shock.

**Figure 3:**
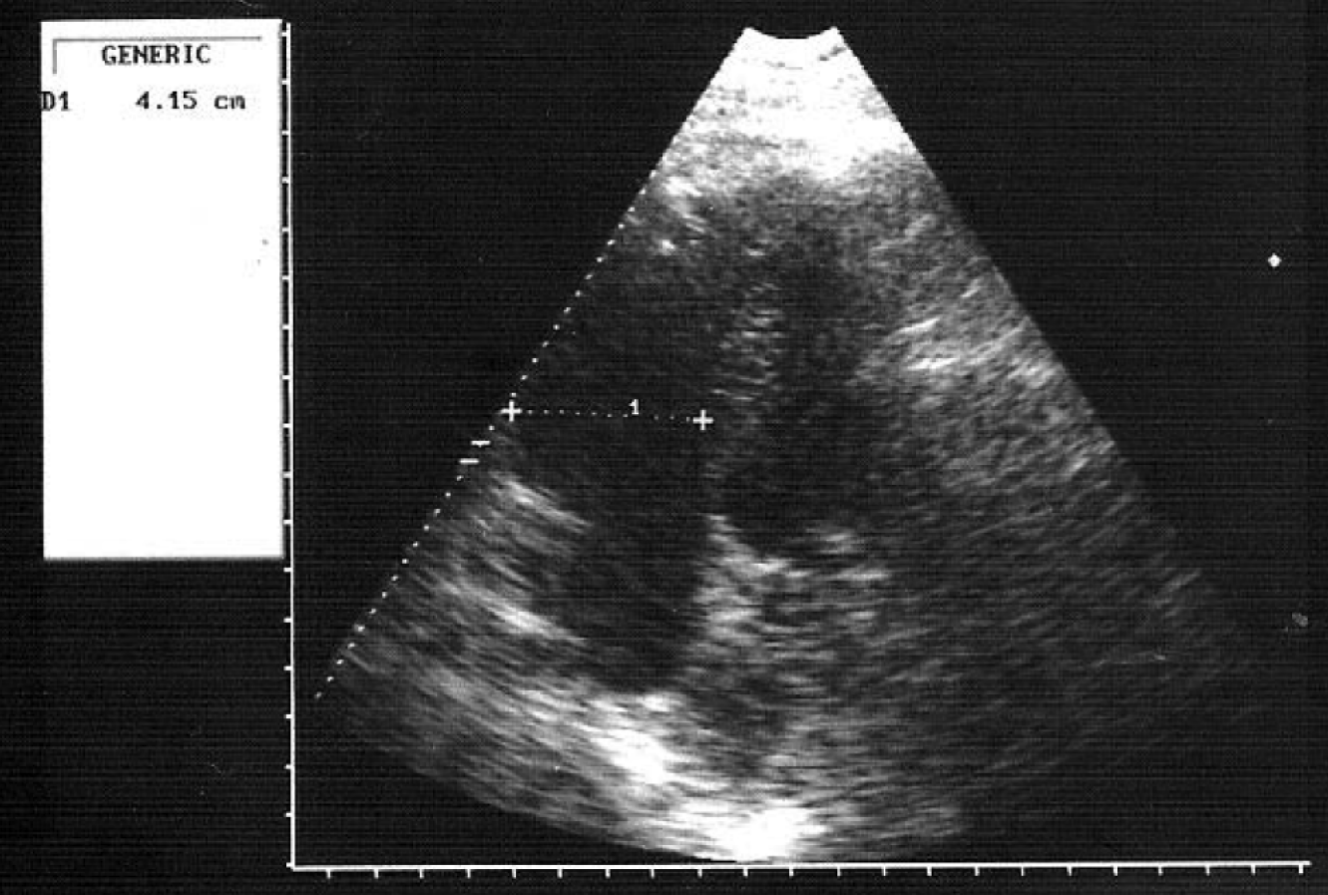
**Apical 4-chamber view revealing a markedly dilated right ventricle with an end-diastolic diameter of 4.15 cm**. There is no evidence of right ventricular or left ventricular thrombus.

Laboratory investigations revealed evidence of myocardial damage with elevation of serial cardiac enzymes and troponin. The kidney functions showed evidence of renal hypoperfusion with a blood urea of 126 mg/dl and serum creatinine of 3.2 mg/dl. Echocardiography was performed and revealed left ventricular systolic dysfunction with an ejection fraction of 35% and a severely hypocontractile right ventricle with a diameter of 4.15 cms in diastole as shown in Figure 3. There was no evidence of a left ventricular or right ventricular thrombus. The bubble test using agitated saline confirmed the absence of a PFO.

**Figure 4:**
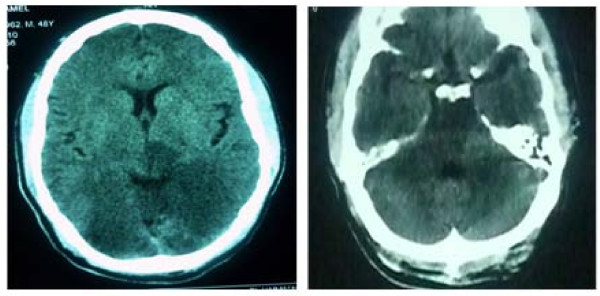
**Revealing hypodense areas in both occipital lobes, brain stem and both cerebellar hemispheres denoting massive infarction in the basilar artery territory**.

On the second hospital day, a follow-up CT brain confirmed the presence of a massive infarction involving the vertebra-basilar territory leading to bilateral occipital associated with brain-stem and cerebellar infarction as shown in figure 4. The patient then succumbed to severe cardiogenic shock resistant to all resuscitative effort.

## Discussion

Concomitant acute myocardial infarction and ischemic cerebrovascular strokes has been rarely reported in the literature [1]. In this report, after the patient has developed acute transmural infero-posterior and right ventricular infarction he suddenly developed a rapid onset and a progressive course of worsening of his conscious level with an NIHSS >25 [2] denoting severe ischemia. This was later confirmed by computed tomography of the brain which revealed massive cerebral and brainstem infarction. We suggest that there is a certain relationship connecting both pathologies together and not just a mere association. We assume that the following mechanisms may explain the occurrence of an ischemic cerebrovascular stroke immediately after an acute inferior and right ventricular infarction.

1- If the underlying pathological mechanism for this transmural myocardial infarction is dissection of the ascending aorta extending to the right coronary ostia, then an extension of the dissection process to involve the vertebral artery which can further extend to involve the basilar artery can explain the association of both events. In our case, the affection of the brain-stem, both cerebellar hemispheres and both occipital lobes indicates that the basilar artery is the culprit vessel for his CNS manifestations.

2- Patients with right ventricular infarction especially those with severely impaired right ventricular function can be complicated with the development of a right ventricular thrombus. In approximately 20% of the population, the foramen ovale fails to seal entirely [3]. A patent foramen ovale (PFO) in a patient with right ventricular failure and subsequent elevation of right sided pressure can be a cause for paradoxical embolism after development of right ventricular thrombus.

3- The sudden development of severe hypotension in patients with long standing hypertension and initial failure of the auto-regulatory mechanisms can lead to sudden reduction in the cerebral blood flow and subsequent infarction. This is especially evident in the brainstem more than the rest of the brain where there is relative inability of the brainstem circulation to auto-regulate intravascular pressures. In the Systolic Hypertension in the Elderly Program (SHEP), which demonstrated an overall benefit of antihypertensive therapy in the prevention of strokes [4], lowering of diastolic blood pressure to less than 60 mm Hg was paradoxically associated with an increased risk of stroke [5]. Severe hypotension may complicate acute right ventricular infarction and can explain this massive cerebral infarction.

4. Ischemia at a distance is another possibility that can explain the left ventricular systolic dysfunction present in this patient despite a small infarction. In a patient with a previously atherosclerotic left anterior descending artery and is dependant mainly on the right coronary artery as a source of collateral circulation supplying the left ventricle; if the RCA is totally occluded, then this will dramatically affect the left ventricular function. Left ventricular systolic dysfunction can be complicated by the formation of a left ventricular thrombus and subsequent embolization. In our patient, the preserved R waves in the precordial leads suggest a previously non-infarcted left ventricle. The only possible explanation for the left ventricular dysfunction on the echocardiogram is "ischemia at a distance".

Due to the rapid downhill course of the disease process in our patient, we could not radiologically confirm the exact underlying pathophysiologic mechanism explaining this association. However, the absence of echocardiographic evidence of a right or left ventricular thrombus and PFO makes thrombo-embolism a remote possibility. Also, the diameter of a patent foramen ovale is usually small which will not permit the passage of a large thrombus sufficient to cause a large infarction as described in the case. Therefore we assume that the most likely explanation to the concomitant cardiac and cerebral infarction in our case is either a dissection that first obstructs the right coronary artery followed subsequently by its extension and obstruction of the basilar artery, or that severe stenosis in the vertobro-basilar territory existed prior to the present episode, and a furthur decrease of blood flow in this area following a marked reduction of blood pressure after right ventricular infarction resulted in the development of massive cerebral infarction.

This case was such a complicated one that carried an unfavorable prognosis as it comprised two pathologies both of which can be life-threatening. The report aims only to provide possible explanations for this cardio-cerebral infarction and to sensitize readers to this association which needs further research. The lack of guidelines that describe the ideal management strategies further complicates the situation. Whether Alteplase therapy is beneficial for concomitant acute myocardial infarction and acute ischemic stroke should be studied. Further studies are also warranted to determine whether it is better to close the PFO or not in patients with high risk of thrombo-embolic complications.

## Abbreviations

PFO: patent foramen ovale; CPK: creatine phosphokinase; CK-MB: creatine kinase MB; GCS: Glasgow coma score; NIHSS: National Institute of Health Stroke Scale; CT: computed tomography; RCA: right coronary artery.

## Competing interests

The authors declare that they have no competing interests.

## Authors' contributions

HO and AF were responsible for drafting the manuscript. RR and EH have made critical revisions to the manuscript. All authors have read and approved the final manuscript.

## Consent

Written informed consent was obtained from the patient's next of kin for publication of this case report. A copy of the written consent is available for review by the Editor-in-Chief of this journal
